# Image-Based Telecom Fraud Detection Method Using an Attention Convolutional Neural Network

**DOI:** 10.3390/e27101013

**Published:** 2025-09-27

**Authors:** Jiyuan Li, Jianwu Dang, Yangping Wang, Jingyu Yang

**Affiliations:** School of Electronic and Information Engineering, Lanzhou Jiaotong University, Lanzhou 730070, China; 13519311970@163.com (Y.W.); yangjy@mail.lzjtu.cn (J.Y.)

**Keywords:** telecommunication fraud detection, convolutional neural network, feature generation

## Abstract

In recent years, telecom fraud remains prevalent in many regions, severely impacting people’s daily lives and causing substantial economic losses. However, previous research has mainly relied on expert knowledge for feature engineering, which lags behind and struggles to adapt to the continuously evolving patterns of fraud effectively. In addition, the extreme imbalance in fraud amounts within real communication data hinders the development of deep learning methods. In response, we propose a feature transformation method to represent users’ communication behavior as comprehensively as possible, and develop a convolutional neural network (CNN) with a Focal Loss function to identify rare fraudulent activities in highly imbalanced data. Experimental results on a real-world dataset show that, under conditions of severe class imbalance, the proposed method significantly outperforms existing approaches in two key metrics: recall (0.7850) and AUC (0.8662). Our work provides a new approach for telecommunication fraud detection, enabling the effective identification of fraudulent numbers.

## 1. Introduction

While telecommunication networks have played a crucial role in economic and social development, the rapid advancement of mobile communication technologies and the widespread use of smart devices have also made them a major platform for fraud [1,2]. According to statistics, telecom fraud in China has led to significant financial losses, including CNY 35.37 billion in 2020 [3] and over CNY 326 billion intercepted in 2021 [4]. Additionally, more than 370,000 fraud cases were detected in 2021, with over 400,000 cases identified in both 2022 [5] and 2023 [6]. Once these fraud cases occur, it is extremely difficult to recover the funds involved [7]. Thus, there is an urgent need to establish effective prevention and management strategies to tackle telecom fraud.

In response to this growing threat, current fraud detection technologies primarily rely on analyzing users’ communication behaviors and content to develop recognition systems. Traditional detection models [8,9,10] focus on rule engines and blacklists, which trigger detection mechanisms through predefined characteristics of fraudulent behavior. In recent years, research has focused mainly on combining expert knowledge for feature engineering and applying machine learning algorithms to detect fraud, including Random Forests [11,12,13], Support Vector Machines [14,15,16,17,18], ensemble learning [19], and neural networks [20,21,22,23,24]. At the same time, graph-based fraud detection technologies predict fraud by learning the features of user interaction behaviors [25,26,27,28,29]. However, the aforementioned methods largely rely on manually designed features based on expert knowledge. The continuous evolution of fraud tactics [30] makes this reliance on manually crafted features costly and unsustainable. Therefore, exploring efficient methods that can automatically extract practical features has become a critical challenge in the field of fraud detection. Moreover, the current detection system faces a severe class distribution imbalance issue, where the number of fraud numbers accounts for only a small fraction of the number of normal numbers. The model may not sufficiently learn from the minority class samples during training, which can negatively impact the accuracy of identifying fraudulent cases. Although various solutions to the data imbalance problem have been proposed [31], and some studies [32,33,34] have explored the class imbalance problem in the field of credit card fraud, research on the data imbalance in the field of fraud-related number recognition is relatively scarce. This may lead to suboptimal classification performance on real-world datasets.

To overcome the challenges of automatically extracting practical features and handling class imbalance, we design a solution to detect telecom fraud. First, we introduce the Focal Loss function [35] during the training process to address the severe class imbalance between normal and fraudulent numbers. Focal Loss increases the loss weight of the minority class samples by introducing a balancing factor, while simultaneously reducing the loss weight of easily classified samples through a modulating factor. This enables the model to focus more on the hard-to-classify fraudulent number samples, thereby improving its ability to learn from minority classes. Next, we propose a feature transformation mechanism to effectively capture users’ communication behaviors for fraud detection. Our main contributions are shown below.

(1) To address the challenge of automatically extracting useful features from telecom data, we propose a feature transformation mechanism that converts Call Detail Record (CDR) text data into structured matrices. This mechanism transforms key features into image-like matrices, such as the proportion of call duration per caller and the number of called numbers, capturing the temporal and behavioral patterns of user interactions. These matrices are then stacked together to form an 8-dimensional tensor, which serves as a rich, high-dimensional representation of the user’s communication behavior. By using this transformation, our approach not only automates the feature extraction process but also significantly reduces the need for manual intervention from domain experts.
(2) We propose a novel approach that combines Squeeze-and-Excitation (SE) blocks [36] with a Convolutional Neural Network (CNN) for detecting telephone fraud. The SE blocks dynamically learn a set of weights that enable the model to emphasize the most informative features while suppressing less relevant ones. This adaptive adjustment of channel importance enhances the model’s ability to focus on critical features, improving performance on complex tasks like fraud detection. By incorporating SE blocks into the CNN, our method strengthens the network’s feature selection process, leading to more accurate and reliable fraud detection outcomes.

The rest of the paper is organized as follows: Section 2 briefly introduces related research works on telecommunication fraud detection, Section 3 presents explicit details about our proposed solution, Section 4 describes and analyzes the experimental results, and Section 5 concludes and discusses future work.

## 2. Related Work

Telecom fraud detection has evolved significantly over time, with research efforts spanning rule-based methods, traditional machine learning, deep learning, and more recently, graph neural networks.

### 2.1. Rule-Based Methods

Early anti-fraud technologies primarily relied on rule-based approaches, informed by insights gained from previous telecommunication fraud detection efforts. These insights were translated into rule systems and deployed for real-time detection, enabling user identification. Taniguchi et al. [9] proposed a rule-based method that combines customer and behavioral data, using a greedy algorithm and adjusted thresholds to select an optimal rule set. However, as the volume of communication services has increased, telecom user data has grown, and fraud techniques have continued to evolve, rule-based anti-fraud methods are insufficient to meet the demands of modern telecommunication fraud detection. Consequently, researchers have increasingly turned to machine learning and deep learning techniques to address these challenges.

### 2.2. Traditional Machine Learning

As an intuitive approach, manually designed features combined with classical machine learning algorithms are commonly used to classify fraudulent phone numbers. With the help of various features and machine learning algorithms, many studies have attempted to address the fraud detection problem. Machine learning techniques primarily utilize methods such as Support Vector Machines (SVM) and Random Forest (RF) for the identification and classification of fraudulent activities. Subudhi et al. [18] presented an approach based on the Quarter-Sphere Support Vector Machine (QS-SVM), where they constructed a user behavior profile by comparing a user’s current calling pattern with historical usage patterns. Experimental results demonstrated that QS-SVM achieved more accurate fraud detection and significantly reduced the false alarm rate, outperforming traditional SVM methods. Bai et al. [13] introduced a fraudulent phone call identification model based on Random Forest. This model identifies fraudulent calls by extracting features of fraudulent calls, selecting model variables, and establishing the model. A real-time shutdown system was also constructed. Subudhi et al. [37] proposed a fraud detection method based on the fuzzy C-means clustering algorithm. The algorithm performs clustering analysis by comparing a user’s recent call activities with typical call behaviors, achieving an accuracy of 93.05%. These methods are more flexible compared to traditional rule-based methods and can learn more complex behavioral patterns of users. However, feature engineering still requires specialized knowledge and manually designed features may not be comprehensive enough to respond promptly to the constantly evolving fraudulent characteristics.

### 2.3. Deep Learning Approaches

In recent years, the development of deep learning technology has brought new opportunities for telecom fraud detection. As an algorithm that simulates the human brain’s neural network, deep learning possesses powerful pattern recognition and learning capabilities. Researchers can utilize deep learning models, such as Convolutional Neural Networks (CNNs) and Recurrent Neural Networks (RNNs), to extract high-level features and perform fraud detection. Xing et al. [38] compared the performance of three deep learning models—CNN, LSTM, and Stacked Denoising Autoencoder (SDAE)—with the traditional Random Forest model on call detail record datasets. The deep learning models achieved accuracy rates exceeding 99%. Gowri et al. [20] utilized an algorithm based on Recurrent Neural Networks (RNNs) for detecting telephone spam and scams, without relying on the telephone network infrastructure. By analyzing historical spam call datasets, they achieved a malicious call detection rate and binary call accuracy of over 90%. Li et al. [24] proposed a method based on breadth learning and dual-channel convolutional neural networks (BLS-DCCNN) for identifying fraudulent calls in the context of telecommunications anti-fraud scenarios. This method first generates and enhances features through a breadth learning system (BLS), then combines a dual-channel convolutional neural network (CNN) structure to extract global and local features, respectively, thereby improving high-dimensional feature expression and classification capabilities. Notably, the authors addressed the issue of extreme imbalance between positive and negative samples in real-world data by introducing the Focal Loss loss function during model training. Zhen et al. [21] proposed an approach called CDR2IMG, which transforms a user’s calling and called relative times by time dimension into an image-like feature matrix containing only 0, 1, and −1. The study used CNN and achieved superior performance with an F1-score of 89.98% and AUC of 92.93%. Bernardo et al. [23] proposed a real-time telecom fraud detection system using a combination of Neural Factorization Machines (NFM) and Autoencoders (AE). Their method models customer calling patterns and adapts to changing behaviors with a memory module, outperforming traditional methods with a high AUC of 91.06%, TPR of 91.89%, and F1-score of 95.45%.

### 2.4. Graph Neural Networks

While the above methods can detect fraudulent activities to some extent, most of them are unable to capture the interactive information between users. Recently, prediction models based on graph neural networks have emerged, which can perform fraud detection by learning the hidden features of social networks. Hu et al. [25] developed an end-to-end telecom fraud detection framework named Bridge to Graph (BTG), which effectively addresses the fraud detection challenge in sparsely connected data through graph neural networks. Experimental results showed that BTG significantly outperforms traditional methods in several metrics. On a real-world CDR dataset, BTG achieved an AUC of up to 92.45% and an F1-score of 85.21%. Hu et al. [39] proposed GAT-COBO, a cost-sensitive graph neural network (GNN) model that addresses the graph imbalance problem in telecom fraud detection by combining Graph Attention Networks with ensemble learning, demonstrating improved detection performance on imbalanced datasets. While traditional telecom fraud detection often relies on single-network data, Ren et al. [26] designed a multi-network latent collaborative graph fraud detection model. The model effectively captures heterogeneity in telecom fraud by fusing individual dynamic behaviors and multi-network embedding in voice and SMS networks. Wu et al. [29] introduced LSG-FD, a telecom fraud detection model that leverages latent synergy graph learning to capture fraudster behaviors and tackle graph disassortativity, demonstrating superior performance on real-world datasets like Sichuan, Yelp, and Amazon.

Although the above works addressed fraudulent call detection to some extent, most of them heavily rely on feature engineering, which cannot adapt to the fast-changing modes of fraudsters. In view of this, we propose a data transformation scheme that turns CDR data into image-like matrices and then stacks them into an 8-dimensional tensor.

## 3. Materials and Methods

### 3.1. Datasets

To ensure the model’s effectiveness in real-world applications, we used communication data from a specific city for a complete month. The dataset comprised 93,293 normal call samples (negative instances) and 517 suspected fraud cases (positive instances). From this collection, we randomly selected 10,000 negative samples and all 517 positive samples to construct the experimental subset. The experimental data was divided into a training set and a test set at a 4:1 ratio.

### 3.2. A Fraud Detection Framework

**Figure 1:**
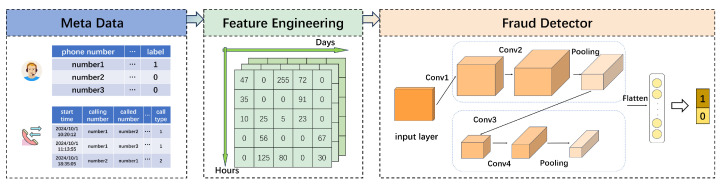
Flowchartof the proposed framework.

We construct eight image-like matrices that integrate behavioral features with the temporal dimension to represent communication behaviors, which are subsequently analyzed using a convolutional neural network. This framework enables the model to gain a comprehensive understanding of communication patterns, thereby enhancing its fraud detection performance. Our proposed framework is shown in Figure 1. In the following section, we introduce each component of the framework in detail.

#### 3.2.1. Feature Engineering

The features are extracted from voice call data. In contrast to the communication behavior features used in previous studies, we introduce two new features. The first, RatioImeichange, represents the frequency with which a user changes terminals. The calculation formula is as follows:

(1) $$\mathrm{RatioImeichange}=\frac{\mathrm{Number}\;\mathrm{of}\;\mathrm{IMEI}\;\mathrm{changes}\;\mathrm{within}\;\mathrm{one}\;\mathrm{hour}}{\mathrm{Total}\;\mathrm{number}\;\mathrm{of}\;\mathrm{IMEI}\;\mathrm{changes}\;\mathrm{within}\;\mathrm{one}\;\mathrm{month}}$$

The second feature, StdRelaAttribution, refers to the standard deviation of the difference between the calling and called attribution codes. StdRelaAttribution reflects the degree of change in the relative position between the calling party and the called number when the user acts as the caller over a period of time, as shown below:

(2) $$\;\mathrm{StdRelaAttribution}\;=\mathrm{std}\left(\left|A_{i}-B_{i}\right|\right)$$

where $\left(A_{i}\right)$ and $\left(B_{i}\right)$ represent the attribution codes of the phone number and its counterpart in the (i)-th call record, and the calculation is performed over all records within one hour in which the phone number serves as the primary call party. In addition, we retain six features commonly used in existing research: proportion of outgoing call duration, proportion of incoming call duration, proportion of outgoing call count, proportion of incoming call count, number of unique counterpart numbers (from outgoing call records), and number of counterpart cities (from outgoing call records).

Inspired by the CDR2IMG approach [21] discussed in Section 2, we create a two-dimensional feature matrix for each feature to describe communication behavior. The *x*-axis of the matrix corresponds to the date, while the *y*-axis represents the hour. We select eight features, including the two newly introduced ones, and aggregate the data within each hour, transforming the values into feature matrices. These eight feature matrices are then stacked along the spatial dimension and used as inputs to a Convolutional Neural Network (CNN), with each matrix corresponding to a distinct dimension in the feature space. This approach allows us to effectively capture and analyze users’ communication behavior in a structured and coherent manner.

#### 3.2.2. Convolutional Neural Network

**Figure 2:**
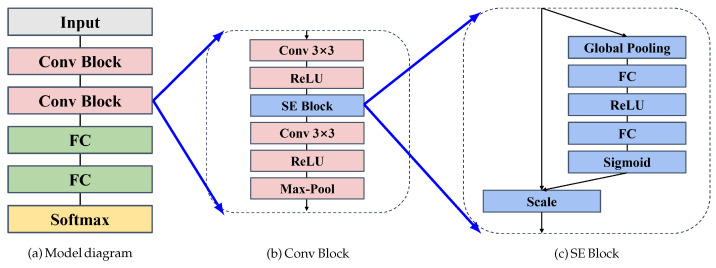
The proposed model in telecommunication fraud detection.

We propose a Convolutional Neural Network (CNN) that integrates Squeeze-and-Excitation (SE) blocks for enhanced feature selection in detecting telephone fraud, as shown in Figure 2. The SE blocks are placed after the activation layers in the convolutional blocks and serve as channel-wise attention mechanisms. They operate by first applying global average pooling to capture global contextual information, followed by fully connected layers that generate channel-specific weights to recalibrate the importance of each feature map. These weights help the network focus on more informative features while suppressing irrelevant ones, thereby improving its ability to detect subtle patterns associated with fraud. By incorporating SE blocks, the model effectively enhances feature selection, leading to better performance on complex classification tasks without significantly increasing computational complexity. This approach allows the network to adaptively prioritize important features, making it more robust and accurate for tasks such as fraud detection. The kernel sizes and filter counts used in our method were varied, and their detailed configurations are provided in Table 1.

Furthermore, to address the severe class imbalance between the proportion of normal numbers and fraudulent numbers in the dataset, we introduce the Focal Loss function. Fraudulent number detection can be viewed as a binary classification problem. In the case of class imbalance, the cross-entropy loss is easily influenced by samples from the majority class. A large number of easily classified normal number samples dominate the gradient updates, making it difficult for the model to effectively learn the characteristics of fraudulent samples. Focal Loss adjusts the weights of the loss function to reduce the loss contribution of easily classified samples, thereby focusing the model on the hard-to-classify fraudulent samples during training. It has the following expression:

(3) $$FL\left(p_{t}\right)=-\alpha_{t}{\left(1-p_{t}\right)}^{\gamma }\log\left(p_{t}\right)$$

(4) $$\alpha_{t}=\left\{\begin{array}{c} \alpha ,\;\;\mathrm{if}\;\;y=1\\ 1-\alpha ,\;\;\mathrm{otherwise}\; \end{array}\right.$$

(5) $$\;p_{t}=\left\{\begin{array}{c} \;p,\;\;\mathrm{if}\;\;y=1\\ 1-\;p,\;\;\mathrm{otherwise}\; \end{array}\right.$$

## 4. Experiment and Discussion

### 4.1. Experiment Setup

#### 4.1.1. Training Environment

The training process was executed on a computer with Windows 10, using Python 3.10 and Pytorch 2.4. The following hardware was used: Gen Intel^®^ Core^TM^ i5-12400F (Intel Corporation, Santa Clara, CA, USA) 2.50 GHz and AMD Radeon RX 6750 GRE (Advanced Micro Devices, Inc., Santa Clara, CA, USA). In the training, the batch size was set to 8, with 100 training epochs. The Adam optimizer was used to update the network parameters, with a learning rate of 0.0001 and a weight decay of 1 $\times \;10^{-5}$. In this paper, the weight factor of the Focal Loss function was set to 0.95, and the modulation factor was set to 3.

#### 4.1.2. Parameter Settings

In the experiments, we tested weight decay values of 1 $\times \;10^{-3}$, 1 $\times \;10^{-4}$, and 1 $\times \;10^{-5}$. The parameter α in the focal loss was set based on the ratio of positive to negative samples in the dataset, and we evaluated γ values of 0, 1, 2, 3. The hyperparameter set that achieved the highest performance was selected as the final configuration. For SVM, RF, and XGBoost, we conducted hyperparameter optimization via grid search to identify the best settings based on recall metrics; detailed results are provided in Table 2. In addition, we tuned the decision threshold (0.496) to maximize recall for our proposed method.

#### 4.1.3. Evaluation Metrics

This section provides an overview of the performance metrics used to evaluate binary classification problems. The predictive performance of classification models is typically evaluated using metrics such as precision, recall, accuracy, AUC, and F1-score. The calculation of these metrics is primarily based on the confusion matrix, which serves as the foundation for computing the average performance metrics of the model. Table 3 shows a confusion matrix for a binary classification model.

Accuracy, which is the proportion of users predicted correctly out of the total number of users, generally indicates better model performance with higher values. However, in the presence of class imbalance, the accuracy tends to favor the class with a larger number of samples (in this case, non-fraudulent users).

(6) $$\;\mathrm{Accuracy}\;=\frac{TP+TN}{TP+TN+FN+FP}$$

Precision refers to the proportion of samples predicted as fraudulent that are actually fraudulent. It quantifies the cost associated with misclassifying non-fraudulent users as fraudulent.

(7) $$\;\mathrm{Precision}\;=\frac{TP}{FP+TP}$$

Recall represents the proportion of actual fraudulent users correctly identified by the model. It signifies the model’s sensitivity in detecting fraudulent users.

(8) $$\;\mathrm{Recall}\;=\frac{TP}{FN+TP}$$

The F1-score is the harmonic mean of the model’s precision and recall, providing a balance between these two metrics.

(9) $$F1\text{-}\mathrm{score}=2\times \frac{\mathrm{Precision}\times \mathrm{Recall}}{\mathrm{Precision}+\mathrm{Recall}}$$

The ROC curve plots the false positive rate (FPR) on the *x*-axis and the true positive rate (TPR) on the *y*-axis. The FPR represents the proportion of normal users incorrectly classified as fraudulent, while the TPR corresponds to the recall. The Area Under the Curve (AUC) quantifies the overall performance of the classifier. For imbalanced datasets, AUC serves as an effective evaluation metric, representing the probability that a randomly chosen positive sample receives a higher prediction score than a randomly chosen negative sample. A higher AUC value indicates more reliable classification performance.

### 4.2. Experimental Analysis

#### 4.2.1. Performance Comparisison

**Figure 3:**
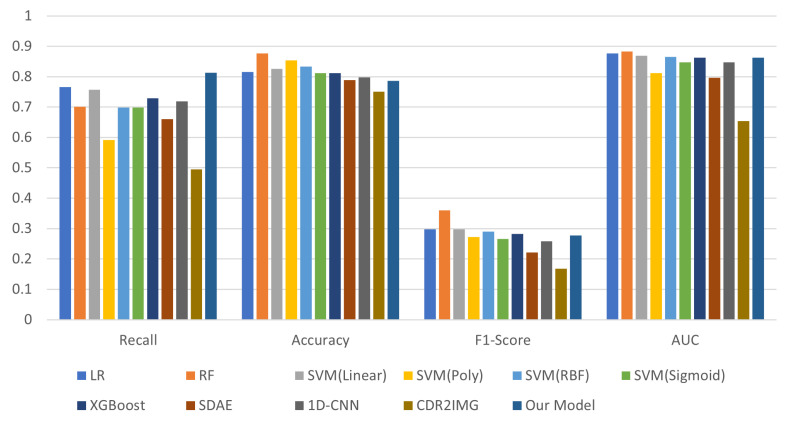
Comparison of different models.

To validate the effectiveness of our model, we compared it against logistic regression (LR), support vector machine (SVM), random forest (RF), Stacked Denoising Autoencoder (SDAE), XGboost, one-dimensional convolutional neural network (1D-CNN), and CDR2IMG based on evaluation metrics. The feature dimension of the pre-processed data used in this study was 44, and the total number of samples was 10,517. Both the 44 tabular features and the 8 map channels were derived from the original data fields through data processing. Specifically, all features used for model inputs were constructed based on the raw communication records and relevant attributes. Among them, the 44 tabular features were obtained through comprehensive preprocessing while the 8 map channels used in the image-based models were further refined from these 44 features by merging, removing redundancy, and selecting the most informative attributes with expert guidance. The SDAE, 1D-CNN, CDR2IMG, and the proposed model employed the Focal Loss cross-entropy loss function. The comparison results are presented in Table 4, and Figure 3 illustrates the comparative performance in recall, accuracy, F1-score, and AUC.

In the domain of fraudulent number identification, given that the cost of missed detections significantly outweighs that of false positives, it is imperative to maximize the coverage of potentially fraudulent numbers. Therefore, recall is the primary metric for evaluating model performance. Distinct performance variations were noted across the evaluated models, as reflected in their Recall, Accuracy, F1-score, and AUC metrics. For example, although Random Forest (RF) achieved the highest Accuracy (0.8763) and F1-score (0.3606), its Recall (0.7009) was lower than that of Logistic Regression (LR) (0.7664) and our proposed model (0.8130). Among the SVM variants, kernel choice led to notable differences. SVM (Linear) attained the highest Recall (0.7573) among SVMs, demonstrating strong performance in fraud detection. Conversely, SVM (Poly) exhibited substantially lower Recall (0.5922), highlighting its limitations in identifying fraud. SVM (RBF) and SVM (Sigmoid) shared the same Recall (0.6990); however, SVM (RBF) outperformed in Accuracy and F1-Score, illustrating the RBF kernel’s advantage in balancing detection and overall classification. LR also delivered competitive results with a Recall of 0.7664 and an AUC of 0.8768, underscoring its reliability in fraud identification.

In contrast, models such as SDAE and CDR2IMG performed poorly across most metrics—CDR2IMG, for instance, recorded the lowest Recall (0.4953) and AUC (0.6535). Notably, although the CDR2IMG model reported outstanding performance metrics in the original literature, its Recall on the experimental dataset was only 0.4953, with an Accuracy of 0.7509, failing to meet expectations. The 1D-CNN approach outperformed CDR2IMG with a Recall of 0.7184, but still lagged behind the model proposed in this paper. It should be noted that both the data distribution and input duration for CDR2IMG in our experiments differed from the original study: while the original work used a six-month dataset with a positive-to-negative ratio of about 1:2, our dataset was based on real-world scenarios with a much lower fraud rate and covered only one month. These differences, particularly the more realistic data and limited duration, may have limited the model’s capacity to capture long-term patterns. However, our design enabled a more comprehensive representation of user behaviors, contributing to the improved effectiveness of our proposed method. Although the classification accuracy of the SDAE was essentially on par with that of the 1D-CNN, its Recall reached only 0.6601. The detection framework introduced in this study achieved a Recall of 0.8130, representing at least a 4.7% improvement over the best model, thereby fully demonstrating its discriminative advantage in fraud detection.

**Figure 4:**
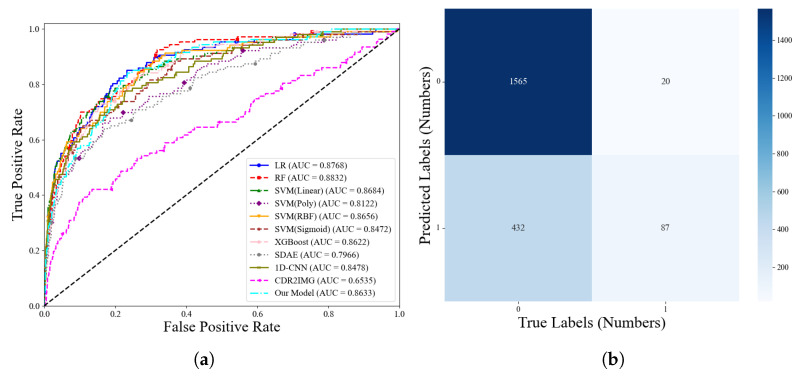
Model Performance Comparison. (**a**) ROC Curves of Different Models; (**b**) Confusion Matrix of Our Proposed Model.

The performance of our model and its comparison models in terms of ROC curves and AUC values is shown in Figure 4. As illustrated in the figure, our model achieved the highest AUC, reaching a value of 0.8632. To provide a more detailed presentation of the classification results for fraudulent number identification using our proposed model, the corresponding confusion matrix is shown in Figure 4.

**Figure 5:**
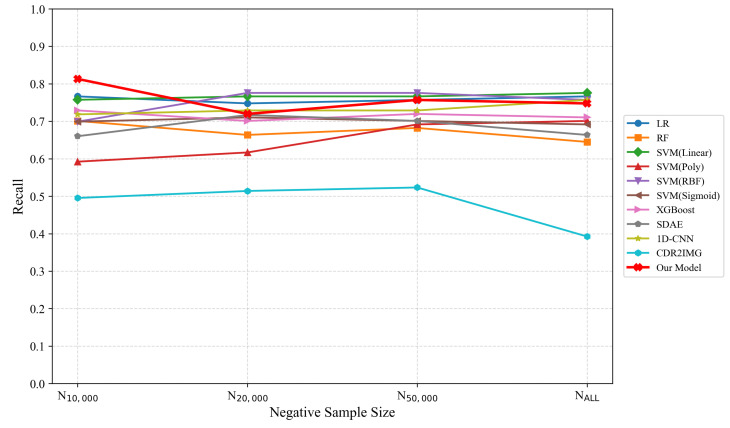
Recall with Different Numbers of Negative Samples.

In addition, to comprehensively evaluate the impact of negative sampling on model performance, we conducted experiments under both the original class prevalence (0.55%) using the full dataset and under different negative sample sizes. The results are presented in Table 4 and illustrated in Figure 5. Compared with the 10,000 negative sample setting used in the main experiments, most methods showed an increase in recall on the full dataset, except for RF, XGBoost, CDR2IMG, and our proposed method. Although some models achieved a maximum recall of 0.7757 under different negative sampling rates, this value is still lower than the recall of our method (0.8130) with the 10,000-negative-sample setting.

To further verify the practicality of our method in real-world scenarios, we also conducted a lightweight performance evaluation on a Windows PC equipped with a single AMD Radeon RX 6750 GRE (Advanced Micro Devices, Inc., Santa Clara, CA, USA). For this experiment, 1000 samples were randomly selected from the test dataset with the batch size set to 1. The measured average inference latency was 0.58 ms per sample and the peak GPU memory usage was 12 MB. This result confirms that our approach not only achieves superior detection performance but also fully satisfies the requirements for real-time online deployment.

A comprehensive evaluation of the three key metrics—Accuracy, Recall, and AUC—demonstrates that the model proposed in this paper offers significant advantages in overall performance. The model substantially improves the identification rate of fraudulent telephone users while maintaining a high level of classification accuracy.

#### 4.2.2. Ablation Study

**Figure 6:**
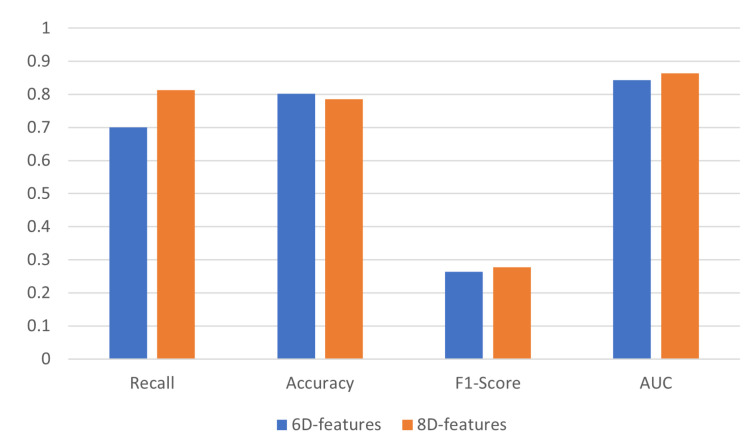
Comparison of Different Features.

To verify the effectiveness of the two newly designed features in this paper, we conducted an ablation study on the dataset. The results, shown in Table 5, present the performance of the CNN model under different feature configurations. The model that excludes the RatioImeichange and StdRelaAttribution features achieved a recall of 0.7009. In contrast, incorporating both features increased the recall to 0.8130, resulting in an absolute improvement of 11.21%. Although the addition of these features led to a slight decrease in accuracy (down by 1.59%), the model’s F1-score and AUC improved, rising by 1.34% and 1.95%, respectively. These findings indicate that integrating RatioImeichange and StdRelaAttribution significantly enhances recall and, when used together, improves overall model performance. Figure 6 illustrates the performance metrics for the different feature configurations. Furthermore, we incorporated two base station-related features into the original 8-dimensional set, resulting in a 10-dimensional feature vector. As shown in Table 5, this expansion did not lead to performance gains; the recall decreased by 10.28% compared to the 8-dimensional configuration.

To better understand the contribution of the focal loss, we replaced the focal loss with weighted cross-entropy loss in our ablation experiment. For the Weighted Cross-Entropy (WCE) loss, experiments were conducted on the dataset with 10,000 negative samples. The positive-to-negative class weight ratio was directly computed from the class distribution of this dataset. The results of this setting are reported in Table 5 (WCE). As shown, our proposed method with focal loss achieves better performance than the weighted cross-entropy baseline, demonstrating the effectiveness of the focal loss design in our approach.

## 5. Conclusions and Future Works

In this study, we propose a telecommunication fraud detection method based on real-world data from a northwest city in China. We design features related to communication patterns, and an ablation study confirms that these features enhance the model’s performance. To address the issue of imbalanced samples, we employ the Focal Loss function to adjust the loss weights between positive and negative samples. Experiments on a real-world dataset demonstrate that our method achieves superior overall performance in detecting fraudulent telephone activities.

This study is limited to a single month of data from one region, which prevents evaluation of the model’s temporal and regional generalization. We plan to conduct longitudinal experiments on larger, multi-region datasets to assess temporal stability and adaptability to evolving fraud behaviors. In the current setup, feature matrices were constructed using the entire month’s data (24 h × 31 days), which restricts chronological train–test splits and may lead to information leakage. Future work will redesign the feature extraction pipeline to enable such splits for a more realistic robustness assessment. In addition, we will explore richer and more diverse behavioral descriptors—including fine-grained temporal dynamics and social connectivity patterns—to expand the dimensionality of the feature vectors and enhance their representational capacity. Developing sustainable and adaptive detection mechanisms to keep pace with changing communication patterns will be a priority. Beyond automated feature extraction from raw CDR data, integrating relational information from communication networks and incorporating supplementary data sources may yield more comprehensive behavioral representations and improve feature correlation modeling [40,41].

## Figures and Tables

**Table 1:** The proposed CNN architecture and its parameters.

Layer	Number of Kernels	Kernel Size	Stride	Padding	Output
Input layer	24 × 31 × 8				
Conv1	64	3 × 3	(1, 1)	same	24 × 31 × 64
SE1	64	-	-	-	24 × 31 × 64
Conv2	128	3 × 3	(1, 1)	valid	19 × 26 × 128
Max-pool1	-	2 × 2	(2, 2)	0	9 × 13 × 128
Conv3	64	3 × 3	(1, 1)	same	9 × 13 × 64
SE2	64	-	-	-	9 × 13 × 64
Conv4	128	3 × 3	(1, 1)	valid	4 × 8 × 128
Max-pool2	-	2 × 2	(2, 2)	0	2 ×4 × 128
FC1	-	-	-	-	256 × 1
FC2	-	-	-	-	2 × 1

**Table 2:** Parameter setting table.

Model	Hyperparameters
Our proposed model	Epoch = 100, batch = 8, the optimization method is Adam, learning rate = 0.0001, decay = 1 $\times \;10^{-5}$, α = 0.95, γ = 3
LR	C = 100, penalty = ‘12’
RF	max_depth = 13, max_features = 9, min_sample_leaf = 10, min_samples_split = 50, n_estimators = 200
SVM (linear/poly/RBF/sigmoid)	C = 100, gamma = ‘auto’, cache_ = 500
XGBoost	colsample_bytree = 0.8, gamma = 0, learning rate = 0.01, max_depth = 3, n_estimators = 100
SDAE	Epoch = 800, batch = 512, the optimization method is Adam, learning rate = 0.0001, decay = 1 $\times \;10^{-5}$, α = 0.95, γ = 3
1D-CNN	Epoch = 150, batch = 8, the optimization method is Adam, learning rate = 0.0001, decay = 0.0001, α = 0.95, γ = 3
CDR2IMG	Epoch = 150, batch = 8, the optimization method is Adam, learning rate = 0.0001, decay = 0.0001, α = 0.95, γ = 3

**Table 3:** Confusion Matrix for Binary Classification Model.

User Status	Prediction = 0	Prediction = 1
label = 0	*TN*	*FP*
label = 1	*FN*	*TP*

**Table 4:** Results of different model evaluation metrics under varying numbers of negative samples.

Negative Sampel Count	Metric	LR	RF	SVM (L)	SVM (P)	SVM (R)	SVM (S)	XGBoost	SDAE	1D-CNN	CDR2IMG	Our Model
**N_10,000_**	**Recall**	0.7664	0.7009	0.7573	0.5922	0.6990	0.6990	0.7290	0.6601	0.7184	0.4953	**0.8130**
	**Accuracy**	0.8156	**0.8763**	0.8251	0.8451	0.8327	0.8113	0.8118	0.7882	0.7975	0.7509	0.7859
	**F1-score**	0.2971	**0.3606**	0.2977	0.2723	0.2903	0.2662	0.2826	0.2218	0.2578	0.1682	0.2779
	**AUC**	0.8768	**0.8832**	0.8684	0.8122	0.8656	0.8472	0.8118	0.7966	0.8478	0.6535	0.8632
**N_20,000_**	**Recall**	0.7477	0.6636	0.7664	0.6168	**0.7757**	0.7103	0.7009	0.7169	0.7289	0.5140	0.7196
	**Accuracy**	0.8225	**0.8880**	0.8232	0.8442	0.8293	0.8191	0.8093	0.8093	0.8397	0.7138	0.8190
	**F1-score**	0.1800	**0.2359**	0.1887	0.1710	0.1915	0.1698	0.1608	0.1381	0.1914	0.0856	0.1714
	**AUC**	0.8775	0.8813	0.8809	0.8221	0.8862	0.8577	0.8693	0.8289	**0.8868**	0.6461	0.8734
**N_50,000_**	**Recall**	0.7570	0.6822	0.7664	0.6916	**0.7757**	0.7009	0.7196	0.7009	0.7289	0.5233	0.7570
	**Accuracy**	0.8241	**0.8849**	0.8272	0.8226	0.8333	0.7720	0.8091	0.8135	0.8206	0.6754	0.8069
	**F1-score**	0.0835	**0.1115**	0.0859	0.0762	0.0897	0.0320	0.0739	0.0736	0.0792	0.0330	0.0766
	**AUC**	0.8755	0.8757	0.8793	0.8357	0.8863	0.8227	0.8618	0.8288	**0.8844**	0.6321	0.8616
**N_*ALL*_**	**Recall**	0.7664	0.6449	**0.7757**	0.7009	0.7570	0.6916	0.7103	0.6635	0.7570	0.3925	0.7476
	**Accuracy**	0.8233	**0.8837**	0.8252	0.814	0.8368	0.7073	0.8089	0.8321	0.8379	0.8066	0.7713
	**F1-score**	0.0471	**0.0595**	0.0482	0.0412	0.0502	0.0262	0.0209	0.0431	0.0505	0.0226	0.0362
	**AUC**	0.8763	0.8777	**0.8816**	0.8339	0.8812	0.7753	0.8505	0.8088	0.8755	0.6064	0.8429

**Table 5:** Ablation study on loss functions and feature dimensions.

Model	Recall	Accuracy	F1-Score	AUC
6d feature model	0.7009	0.8018	0.2645	0.8437
8d feature model (WCE)	0.7757	0.7884	0.2716	0.8436
8d feature model	0.8130	0.7859	0.2779	0.8632
10d feature model	0.7102	0.8174	0.2835	0.8677

## Data Availability

The data presented in this study are available on request from the corresponding author. The data are not publicly available due to privacy restrictions.
